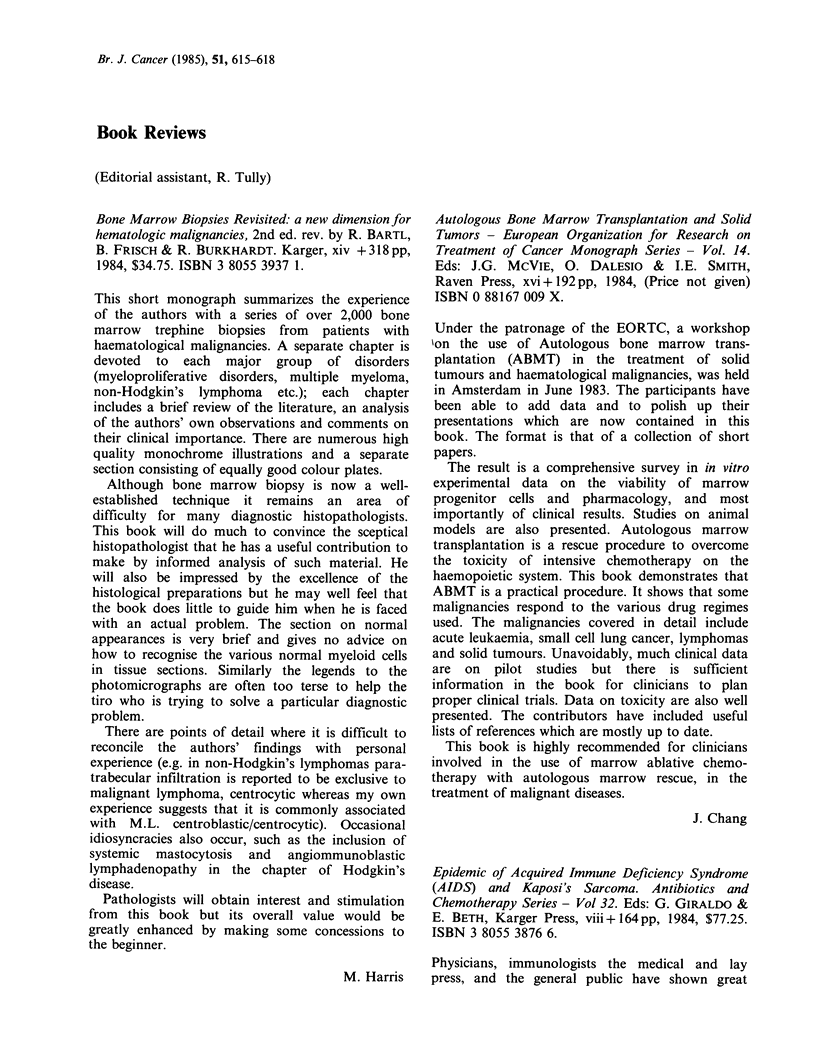# Autologous Bone Marrow Transplantation and Solid Tumors - European Organization for Research on Treatment of Cancer Monograph Series - Vol. 14

**Published:** 1985-04

**Authors:** J. Chang


*Autologous Bone Marrow Transplantation and Solid Tumors – European Organization for Research on Treatment of Cancer Monograph Series – Vol. 14.* Eds: J.G. MCVIE, O. DALESIO & I.E. SMITH, Raven Press, xvi+192 pp, 1984, (Price not given) ISBN 0 88167 009 X.

Under the patronage of the EORTC, a workshop on the use of Autologous bone marrow transplantation (ABMT) in the treatment of solid tumours and haematological malignancies, was held in Amsterdam in June 1983. The participants have been able to add data and to polish up their presentations which are now contained in this book. The format is that of a collection of short papers.

The result is a comprehensive survey in *in vitro* experimental data on the viability of marrow progenitor cells and pharmacology, and most importantly of clinical results. Studies on animal models are also presented. Autologous marrow transplantation is a rescue procedure to overcome the toxicity of intensive chemotherapy on the haemopoietic system. This book demonstrates that ABMT is a practical procedure. It shows that some malignancies respond to the various drug regimes used. The malignancies covered in detail include acute leukaemia, small cell lung cancer, lymphomas and solid tumours. Unavoidably, much clinical data are on pilot studies but there is sufficient information in the book for clinicians to plan proper clinical trials. Data on toxicity are also well presented. The contributors have included useful lists of references which are mostly up to date.

This book is highly recommended for clinicians involved in the use of marrow ablative chemotherapy with autologous marrow rescue, in the treatment of malignant diseases.

J. Chang